# Effects of a Health Promotion Intervention on Physical Activity in African American Men Living with HIV: Randomized Controlled Trial

**DOI:** 10.1089/apc.2021.0039

**Published:** 2021-10-08

**Authors:** John B. Jemmott, Loretta S. Jemmott, Jingwen Zhang, Larry D. Icard, Terri-Ann Kelly, Ian Frank, Scarlett L. Bellamy

**Affiliations:** ^1^ Annenberg School for Communication, University of Pennsylvania, Philadelphia, Pennsylvania, USA.; ^2^ Department of Psychiatry, Perelman School of Medicine, University of Pennsylvania, Philadelphia, Pennsylvania, USA.; ^3^ College of Nursing and Health Professions, Drexel University, Philadelphia, Pennsylvania, USA.; ^4^ Department of Communication and University of California, Davis, Davis, California, USA.; ^5^ Department of Public Health Sciences, University of California, Davis, Davis, California, USA.; ^6^ School of Social Work, College of Public Health, Temple University, Philadelphia, Pennsylvania, USA.; ^7^ Centre for Communication Technologies, School of Information Technology, Nelson Mandela University, Port Elizabeth, South Africa.; ^8^ School of Nursing, Rutgers University, Camden, New Jersey, USA.; ^9^ Department of Medicine, Perelman School of Medicine, University of Pennsylvania, Philadelphia, Pennsylvania, USA.; ^10^ Department of Epidemiology and Biostatistics, Dornsife School of Public Health, Drexel University, Philadelphia, Pennsylvania, USA.

**Keywords:** people living with HIV, health promotion intervention, African American men, aerobic physical activity, muscle-strengthening physical activity, non-communicable diseases

## Abstract

HIV and its treatment with antiretroviral therapy increase the risk of noncommunicable diseases (NCDs) tied to physical inactivity. Older African American men are also at high risk for NCDs. We tested the efficacy of a theory-based intervention to increase adherence to federal aerobic and muscle-strengthening physical activity (PA) guidelines among African American men aged 40 years and older living with HIV. We randomized African American men aged 40 years and older living with HIV to a three-session social cognitive theory-informed health promotion intervention targeting PA or a one-session health awareness control condition. The primary outcome was PA guideline adherence assessed (self-reported) preintervention, immediate postintervention, and 3, 6, and 12 months postintervention. Secondary outcomes were the number of days on which participants reported moderate-intensity aerobic PA, vigorous-intensity aerobic PA, and muscle-strengthening PA in the past 7 days. Of 302 participants, 255 completed the 12-month postintervention measures. Generalized estimated equation logistic regression indicated that the health promotion intervention participants had higher odds of meeting PA guidelines than health awareness control participants, adjusting for baseline adherence (*p* = 0.011). Health promotion intervention participants also reported more muscle-strengthening PA (*p* = 0.001), vigorous-intensity aerobic PA (*p* = 0.049), and moderate-intensity aerobic PA (*p* = 0.010) than control participants. The rise in self-reported adherence to PA guidelines and improvements in muscle-strengthening and aerobic PA considered separately suggest that a relatively brief behavioral intervention can increase PA among African American men aged 40 years and older living with HIV and potentially curb their risk of NCDs that PA can prevent.

## Introduction

Strategies are needed to encourage African American men living with HIV to engage in physical activity (PA) to reduce their risk for and mitigate the effects of noncommunicable diseases (NCDs) associated with HIV, including diabetes mellitus; hypertension; cardiovascular disease; diminished bone density; and sarcopenia, muscle loss, and associated frailty.^1–3^ People living with HIV (PLWH) are surviving longer and suffering fewer AIDS-defining opportunistic infections and cancers because of the success of antiretroviral therapy (ART).^4^ However, considerable evidence suggests that HIV and its treatment with ART can increase the risk for NCDs associated with aging.^1,5^ Thus, rising NCD rates have accompanied the increased life expectancy among PLWH,^6^ which underscores the need to prevent and manage NCDs affecting PLWH.

Further, African Americans have unusually high rates of both HIV and NCDs. For instance, African American men have the highest HIV prevalence among racial/gender subgroups in the United States.^7^ At the end of 2018, the prevalence of HIV was 1762 per 100,000 among African American men compared with 805 among Hispanic/Latino men, 314 among White men, 214 among Native American men, 164 among Asian men, and 798 among African American women.^7^ African American men also have the highest mortality rates from cardiovascular diseases of any racial/gender subgroup in the United States^8^ and higher mortality rates from diabetes than African American women or White men and women.^9^

Although PA reduces the risk of NCDs,^10^ PLWH have low rates of PA,^11^ as do African Americans.^12^ Studies have shown that regular PA is associated with reduced risk of diabetes mellitus and with lower cardiovascular and all-cause mortality rates.^13,14^ Further, low muscle mass is associated with impaired performance of activities of daily living and increased mortality among middle-aged African Americans.^15^ The Department of Health and Human Services (DHHS)-provided guidelines for PA indicate that adults should perform both aerobic PA and muscle-strengthening PA weekly.^16^ However, African Americans are less likely to meet the DHHS PA guidelines. Only 21% of African American adults meet the DHHS guidelines compared with 25% of non-Hispanic White adults.^12^ These findings highlight the need for interventions to encourage PA among African Americans.

Engaging in healthful behaviors, including regular PA, is an essential aspect of lifestyle optimization for PLWH.^17^ Evidence indicates that aerobic and resistance exercises improve cardiorespiratory fitness, strength, and body composition in PLWH.^18,19^ However, recent literature reviews have documented that many PLWH do not meet PA guidelines.^11^ Some evidence suggests that rates of PA among PLWH may be lower than their counterparts not living with HIV.^20^ Moreover, a meta-analysis reported high dropout rates in PA interventions with PLWH.^21^

Only a few trials have tested interventions to help PLWH improve their PA.^22–24^ In this study, we report the results of a randomized controlled trial (RCT) to address this gap in the literature. We tested the efficacy of a theory-based intervention in increasing PA in African American men aged 40 years and older living with HIV. We randomly assigned them to the theory-based intervention or a brief intervention control condition. We hypothesized that the intervention group would be more likely to report adherence to PA guidelines than the control group during the 12 months postintervention, adjusting for baseline adherence.

## Methods

All participants provided written informed consent. Institutional Review Board No. 8 of the University of Pennsylvania approved the study (Protocol No. 813202). Men aged 40 years or older, receiving ART for HIV, and self-identifying as Black or African American were eligible to participate. We excluded men who (1) had a blood pressure of 180/110 mm Hg or higher, (2) reported participating in a health promotion intervention trial targeting PA, diet, or prostate or colon cancer screening in the past 12 months, or (3) did not have a mailing address or planned to move beyond a reasonable distance from the study site within the next 18 months.

We recruited the men from a database of PLWH who consented to be contacted for research studies, through referrals from local HIV service providers and AIDS service organizations, and through ads in a local newspaper and social media sites, including Facebook, Twitter, and Craigslist. We enrolled the participants over 35 months, beginning in January 2015 and completing all data collection by December 2018. We held the intervention and data collection sessions in university conference rooms.

The study used an RCT design. After completing Visit 1 baseline assessments, men were scheduled for Visit 2 for randomization and intervention. Using computer-generated random number sequences, we randomly assigned them in a 1:1 ratio to the Men Together Making a Difference Health Promotion or health awareness control condition. One researcher generated random assignments, and the project director implemented them. Data collectors, but not the facilitators and participants, were blinded to random group assignment.

### Health promotion intervention

The Men Together Making a Difference Health Promotion Intervention is an adaptation (for men) of the Eban Health Promotion Intervention, which increased adherence to DHHS PA guidelines in a multi-site RCT with African American HIV-serodiscordant couples.^25^ Feedback from focus groups with African American men living with HIV helped us revise the intervention to ensure that the activities focused on and emphasized African American men aged 40 years and older living with HIV. Since some activities were designed to be implemented in sessions with individual HIV-serodiscordant couples,^26^ we revised them to be appropriate for groups of men. To reduce costs, we employed a single facilitator instead of the cofacilitators who implemented the Eban intervention.

The intervention based on social cognitive theory^27^ and the reasoned action approach^28^ integrated with formative research was designed to increase attitudes, self-efficacy, and intention supportive of behaviors tied to decreased cardiovascular disease, diabetes, hypertension, and cancer risk. Table 1 presents an outline of the intervention. Brainstorming, educational games, and interactive activities, including physical exercise and videos,^26,29^ were used to increase adherence to guidelines for PA, 5-a-day diet, and colon cancer screening. It encouraged 30 min or more of moderate-intensity aerobic PA on 5 days or 20 min or more of vigorous-intensity aerobic PA on 4 days each week and muscle-strengthening PA on 2 or more days each week. Participants identified their barriers to engaging in the recommended behaviors and strategies for surmounting those barriers.

**Table 1.** Men Together Making a Difference Health Promotion Intervention

Session 1: Looking after you and your body: nutrition
Module 1: Welcome and Black men's health issues
Activity 1A: Welcome and program overview (5 min)
Activity 1B: Group introductions (10 min)
Activity 1C: Creating group guidelines (10 min)
Activity 1D: Health concerns for Black men living with HIV (10 min)
Activity 1E: Introduction of prevention, detection, and control (10 min)
Activity 1F: Assessing your health: health risk assessment (15 min)
Module 2: You are what you eat
Activity 2A: Healthy personal eating video and brainstorming (20 min)
Activity 2B: Food plate guide (10 min)
Activity 2C: 5-A-Day (15 min)
Activity 2D: Identify barriers and find solutions (15 min)
Module 3: Healthy eating strategies and review
Activity 3A: Nutrition family feud review game (15 min)
Activity 3B: Let's make a smoothie (20 min)
Activity 3C: Homework assignment—menu plan (20 min)
Activity 3D: Session closing (5 min)
Session 2: Getting fit and being strong: exercise
Module 4: You are what you do: physical activity for your health
Activity 4A: Welcome back and overview (5 min)
Activity 4B: Homework review—meal plan (10 min)
Activity 4C: Understanding body–mass index (5 min)
Activity 4D: Physical activity assessment (10 min)
Activity 4E: Strength-building exercise video workout (30 min)
Module 5: Exercise and your health
Activity 5A: Barriers and solutions to exercising for Black men age 40 and older living with HIV (15 min)
Activity 5B: Attitudes toward exercise, forced choice (15 min)
Activity 5C: Exercising for your heart: aerobic exercise video workout (30 min)
Module 6: Physical activity strategies and review
Activity 6A: Health Jeopardy (20 min)
Activity 6B: Homework assignment—exercise plan (20 min)
Activity 6C: Session closing (5 min)
Session 3: Managing your future health
Module 7: Early detection and screening
Activity 7A: Welcome back and overview (5 min)
Activity 7B: Homework review—exercise plan (10 min)
Activity 7C: Disease prevention review (10 min)
Activity 7D: Early detection and screening (10 min)
Activity 7E: Colorectal cancer (25 min)
Module 8: Healthy eating and exercise review
Activity 8A: Nutrition review (5 min)
Activity 8B: Top Chef game (15 min)
Activity 8C: Exercise review (15 min)
Activity 8D: Exercise charades (10 min)
Activity 8E: Making a snack—air-popped popcorn (15 min)
Module 9: Pulling it all together—wrap up and review
Activity 9A: Health Basketball (20 min)
Activity 9B: Building support for healthy living (15 min)
Activity 9C: Letter to self (10 min)
Activity 9D: Final session closing ceremony (15 min)

There were nine 1-h modules delivered in three sessions over three consecutive weeks. Sessions 1 and 2 included homework assignments on a healthy diet or PA, and the subsequent session addressed their barriers to completing the assignment and strategies to overcome the obstacles. Participants learned about moderate and vigorous aerobic activity and muscle-strengthening activity, including DHHS guidelines. They learned how to exercise safely using exercise bands and practiced with a muscle-strengthening exercise video depicting three levels of intensity depending on the participant's level of fitness, with the lowest intensity involving exercising while sitting in a chair. They brainstormed barriers to aerobic and muscle-strengthening exercises and generated solutions to the obstacles. Participants received pedometers to monitor their steps and discussed how they could increase their daily steps. They participated in an aerobic exercise workout to music and received an exercise-at-home DVD with both aerobic and muscle-strengthening activities that they could perform independently.

Participants played review games such as Health Jeopardy and Health Basketball to reinforce the information they covered in the curriculum. They discussed the benefits of a support system and identified people in their lives who could be supportive of healthy eating and exercise habits. They wrote a letter to self, promising to practice healthy behaviors, including reasons for engaging in healthy behavior and examples of how they will engage in such behavior. They placed it in a self-addressed envelope to be mailed to them 6 weeks later as a reminder of their commitment. Finally, they participated in a closing ceremony and received a certificate of completion.

### Control condition

The health awareness control condition consisted of one 60-min small-group session led by a trained facilitator. Participants viewed and discussed video clips that encouraged PA, fruit and vegetable consumption, and colon cancer screening.

### Measures

We administered self-report assessments at baseline, immediate postintervention, and 3, 6, and 12 months postintervention using audio computer-assisted self-interviewing (ACASI). We assessed PA using three open-ended questions developed by the Centers for Disease Control and Prevention.^30^ On how many of the past 7 days did you exercise or participate in PA for at least 20 min that made you sweat and breathe hard, such as basketball, soccer, running, swimming laps, fast bicycling, fast dancing, or similar vigorous PAs? On how many of the past 7 days did you exercise or participate in PA for at least 30 min that did not make you sweat and breathe hard, such as walking, slow bicycling, skating, pushing a lawn mower, or anything else that caused small increases in breathing or heart rate? On how many of the past 7 days did you exercise to strengthen or tone your muscles, such as push-ups, sit-ups, or weightlifting?

The a priori primary outcome was adherence to PA guidelines, a binary variable that reflected whether the men reported meeting the guideline of engaging in muscle-strengthening activity on 2 or more days and engaging in either 20 min of vigorous-intensity activity on at least 4 days or 30 min of moderate-intensity activity on at least 5 days in the past 7 days.^16,25^ Later, we created an alternative operationalization of this outcome, which we believe is more accurate, namely a binary measure indicating whether the participant met the criterion of the number of minutes specified in the PA guideline. This new measure (PA minutes guideline met) was also calculated based on the three items, but indicated whether the participant met PA guidelines of engaging in at least 150 min of moderate-intensity PA, 75 min of vigorous-intensity aerobic PA, *or an equivalent combination of moderate- and vigorous-intensity PA* in the past 7 days plus muscle-strengthening PA at least twice in the past 7 days.^16^ Time spent (minutes) in moderate-to-vigorous PA was defined as the sum of the self-reported number of days of moderate-intensity aerobic PA times 30 min and the self-reported number of days of vigorous-intensity aerobic PA times 20 min times two because minutes of vigorous PA count twice as much as minutes of moderate PA.

Secondary outcomes included self-reported days of moderate-intensity aerobic PA, days of intensive aerobic PA, and days of muscle-strengthening PA in the previous 7 days.

Participants also completed measures of sociodemographic characteristics, including age, marital status, education, income, housing stability, employment status, use of alcohol and drugs, and ART adherence. We determined alcohol dependency from a score of 2 or greater on the CAGE questionnaire^31^ and drug dependency from a score of 3 or more on the Texas Christian University Drug Screen.^32^ We defined ART adherence as the proportion of pills taken in the past 3 days.^33^ We also collected measures of PA mediators, healthy diet, healthy diet mediators, anthropometric variables, and colon cancer screening, which we will report in separate articles.

### Sample size and statistical analyses

A priori, we estimated the sample size required to detect a clinically meaningful effect of the health promotion intervention on the primary outcome, adherence to PA guidelines, compared with the health awareness control condition, with adequate power (>80%), accounting for the repeated measures design of our planned longitudinal assessments. An absolute increase of 9.1 percentage points, assuming greater adherence in the health promotion intervention condition, was selected as clinically meaningful. We estimated that the average correlation among PA guideline adherence rates immediate postintervention and 3, 6, and 12 months postintervention was equal to 0.10. Assuming a two-tailed test, α = 0.05, 20% attrition, and a 9.1% increase in adherence to PA guidelines (from 15.7% in the control group to 24.8% in the intervention condition), with 323 men randomized, the estimated statistical power is 87%.

We summarized the participants' baseline sociodemographic characteristics with descriptive statistics and analyzed attrition with chi-square tests and logistic regression analyses. For the primary binary outcome, adherence to PA guidelines, we tested the efficacy of the health promotion intervention compared with the health awareness control condition with logistic generalized estimating equation (GEE) models, with and without adjusting for the baseline of the outcome. We used a similar GEE approach for continuous secondary outcomes by specifying an identity link function. The models included condition, follow-up time (i.e., immediate post-test and 3, 6, and 12 months postintervention), and the outcome's baseline. We used an independent working correlation matrix and robust standard errors. We present efficacy estimates as odds ratios (ORs) or mean differences with corresponding 95% confidence intervals (CIs) to test the hypotheses of interest.

We used an intention-to-treat approach, analyzing participants based on their assigned intervention, irrespective of the number of intervention sessions or postintervention assessments they attended. We used SAS 9.4 for all analyses.

## Results

**FIG. 1.**
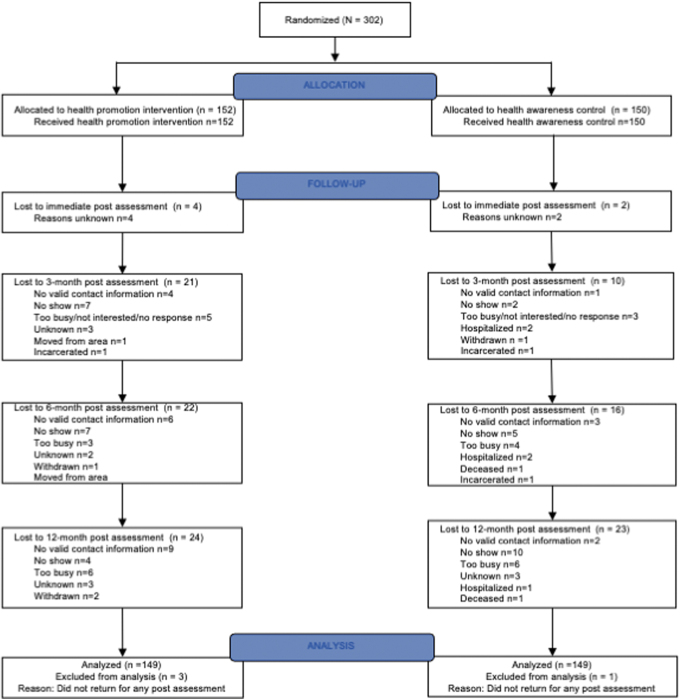
CONSORT flow diagram of the progress of participants through the phases of the trial.

Figure 1 displays the progress of the 302 enrolled participants through the trial's phases. We present the men's sociodemographic characteristics by condition in Table 2. The men ranged in age from 40 to 88 (mean = 53.9; standard deviation = 7.2). Only 50 (17%) were employed and 200 (66%) had less than $850 as monthly income. Forty-four (15%) had unstable housing, 31 (10%) reported alcohol dependency, and 30 (10%) reported drug dependency. Only 34 (11%) met the DHHS PA guideline at baseline.

**Table 2.** Baseline Sociodemographic Characteristics of African American Men Living with HIV by Condition, 2015–2018

Characteristic	Total,* n *(%)	Health promotion,* n *(%)	Health awareness control,* n *(%)
*N*	302	152	150
Age (years), mean (SD)	53.9 (7.2)	53.6 (8.1)	54.2 (6.3)
Married	33/302 (10.9)	22/152 (14.5)	11/150 (7.3)
Employed	50/302 (16.6)	27/152 (17.8)	23/150 (15.3)
Completed high school	228/302 (75.5)	115/152 (75.7)	113/150 (75.3)
Monthly income			
<$ 850	200/302 (66.2)	105/152 (69.1)	95/150 (63.3)
$851 or more	102/302 (33.8)	47/152 (30.9)	55/150 (36.7)
Unstable housing	44/302 (14.6)	27/152 (17.8)	17/150 (11.3)
Alcohol dependent^a^	31/302 (10.3)	18/152 (11.8)	13/150 (8.7)
Drug dependent^b^	30/302 (9.9)	16/152 (10.5)	14/150 (9.3)
Body–mass index, mean (SD)	28.2 (5.9)	27.7 (5.2)	28.7 (6.6)
ART adherence,^c^ mean (SD)	0.9 (0.2)	0.9 (0.2)	0.9 (0.2)

^a^ Determined from a score of 2 or more on the CAGE questionnaire.

^b^ Determined from a score of 3 or more on the Texas Christian University Drug Screen.

^c^ ART adherence indicates the mean proportion of ART pills taken in the past 3 days.

ART, antiretroviral therapy; SD, standard deviation.

The health promotion intervention session attendance was high: 152 (100%) attended session 1 and 148 (97%) attended sessions 2 and 3. All participants in the control condition attended their single intervention session. Postintervention assessment completion was also high, with 296 (98%) completing the immediate post-test and 272 (90%) completing the 3-month, 264 (87%) completing the 6-month, and 255 (84%) completing the 12-month postintervention assessments. The intervention (95%) and control (97%) conditions did not differ in the percentage completing one or more postintervention assessments.

Attending one or more follow-ups was unrelated to baseline adherence to the PA guideline or the sociodemographic variables in Table 2, with one exception. ART adherence predicted attending at least one follow-up. The greater the ART adherence at baseline, the higher the odds of returning for a follow-up, OR = 9.83 (95% CI = 1.55–62.43).

The descriptive statistics on PA outcomes by condition and assessment time are presented in Table 3. The estimated unadjusted and adjusted values for the baseline intervention effects are shown in Table 4. The odds of meeting the PA guideline defined in self-reported days (OR = 1.74; 95% CI = 1.14–2.66) and minutes (OR = 1.88; 95% CI = 1.27–2.77) and self-reported number of days of vigorous-intensity PA (mean difference, 0.29; 95% CI = 0.001–0.58), moderate-intensity PA (mean difference, 0.44; 95% CI = 0.11–0.77), and muscle-strengthening PA (mean difference, 0.40; 95% CI = 0.16–0.64) increased in the health promotion intervention compared with the control condition, adjusting for the outcomes' baseline and assessment time. The unadjusted analyses yielded similar results.

**Table 3.** Self-Reported Physical Activity Outcomes by Condition and Assessment Time for African American Men Living with HIV, 2015–2018

Outcome	Baseline	Postintervention	3 Months	6 Months	12 Months
Meeting the PA guidelines, *n* (%)					
Health promotion intervention	18/152 (11.8)	42/148 (28.4)	31/131 (23.7)	22/130 (16.9)	22/128 (17.2)
Health awareness control	16/150 (10.7)	18/148 (12.2)	22/141 (15.6)	23/134 (17.2)	13/127 (10.2)
Meeting PA guidelines for minutes, *n* (%)					
Health promotion intervention	27/152 (17.7)	63/148 (42.6)	44/131 (33.6)	44/130 (33.9)	34/128 (26.6)
Health awareness control	25/150 (16.7)	28/148 (18.9)	36/141 (25.5)	35/134 (26.1)	23/127 (18.1)
Days of vigorous-intensity PA in the past 7 days, mean (SD)					
Health promotion intervention	1.46 (1.91)	2.47 (1.73)	2.18 (1.96)	2.05 (1.76)	1.91 (1.95)
Health awareness control	1.39 (1.76)	1.90 (1.87)	1.99 (1.91)	1.77 (2.01)	1.72 (1.97)
Days of moderate-intensity PA in the past 7 days, mean (SD)					
Health promotion intervention	2.11 (2.38)	2.86 (2.08)	2.56 (2.16)	2.53 (2.00)	2.30 (2.09)
Health awareness control	2.18 (2.46)	2.22 (2.16)	2.21 (2.21)	2.19 (2.28)	1.91 (2.12)
Days of muscle-strengthening PA in the past 7 days, mean (SD)					
Health promotion intervention	1.11 (1.88)	1.84 (1.78)	1.71 (1.78)	1.55 (1.63)	1.45 (1.78)
Health awareness control	0.99 (1.67)	1.13 (1.63)	1.29 (1.77)	1.23 (1.80)	1.02 (1.43)

PA, physical activity; SD, standard deviation.

**Table 4.** Generalized Estimating Equation Empirical Significance Tests and 95% Confidence Intervals for Intervention Effects on Outcomes of Self-Reported Physical Activity in the Past 7 Days, Adjusted and Unadjusted for Baseline of the Outcome, Among African American Men Living with HIV, 2015–2018

Outcome	Unadjusted for baseline		Adjusted for baseline	
	Estimate (95% CI)	p	Estimate (95% CI)	p
Meeting PA guidelines^a^	1.75 (1.13 to 2.69)	0.011	1.74 (1.14 to 2.66)	0.011
Meeting PA guidelines for minutes^a^	1.85 (1.26 to 2.74)	0.002	1.88 (1.27 to 2.77)	0.002
Vigorous-intensity PA^b^	0.31 (−0.03 to 0.66)	0.074	0.29 (0.001 to 0.58)	0.049
Moderate-intensity PA^b^	0.43 (0.05 to 0.82)	0.028	0.44 (0.11 to 0.77)	0.010
Muscle-strengthening PA^b^	0.48 (0.18 to 0.78)	0.002	0.40 (0.16 to 0.64)	0.001

^a^ Estimate is odds ratio.

^b^ Estimate is mean difference.

CI, confidence interval; PA, physical activity.

There were no adverse events.

## Discussion

The high rates of HIV and NCDs, coupled with low levels of PA adherence, pose a challenge for African American men living with HIV. The present study found that a behavioral intervention increased self-reported adherence to DHHS PA guidelines among African American men aged 40 years and older living with HIV compared with a health awareness control intervention. The intervention also caused significant increases in self-reported aerobic PA and muscle-strengthening PA considered separately. The results are significant because African American men living with HIV commonly do not engage in sufficient PA, and to our knowledge, no other interventions have increased their PA.

The present findings differ from those observed in a recent RCT that also used a behavioral intervention designed to increase PA among a predominantly African American sample of PLWH. The intervention reduced sweetened beverage consumption and weight, but did not increase PA.^24^ The study differed from the present study, in that 30% of the participants were women and they were followed for only 6 months postintervention. A study with South African PLWH found no effect of a 6-month behavioral intervention on pedometer step counts at the 12-month assessment compared with a standard of care control group.^23^ An RCT of a 12-week theory-based PA intervention among older PLHW with functional limitations found that intervention increased PA behavior immediately postintervention compared with a usual care control group.^22^ Our study with up to 12 months of follow-up also supports the efficacy of theory-based behavioral interventions for increasing self-reported PA.

The RCT design is a strength of the study. Considering the meta-analytic evidence^21^ of high treatment dropout in PA interventions with PLWH, the high intervention session attendance and low follow-up assessment attrition are also strengths of this study. It is noteworthy that the intervention was relatively brief, consisting of only three 3-h sessions. Many interventions contain considerably more sessions of face-to-face contact.^34,35^ The present study suggests the possibility that brief interventions may be effective.

The study's limitations include the use of self-reports of PA. Imperfect memory and social desirability responding can diminish the accuracy of self-reported behavior. To reduce such bias, we used ACASI, which decreases socially desirability bias compared with the alternative of paper-and-pencil surveys and face-to-face interviews.^36^ Future research should investigate whether the effects we observed will replicate in a trial using objective PA behavior measures. Another limitation is that the participants were African American men from one city; the findings may not generalize to all African Americans aged 40 years or older living with HIV. Despite these limitations, this study's results have important implications for intervention research among PLWH and provide vital information for programs aimed at improving outcomes for PLWH.

In conclusion, a theory-based behavioral intervention increased self-reported adherence to DHHS PA guidelines during a 12-month follow-up period. This intervention shows promise for uptake, adoption, and evaluation in real-world HIV-serving clinical and community settings using implementation science research strategies. More generally, the findings have implications for interventions to increase daily PA for African American men living with HIV, a population at high risk of NCDs that PA can prevent. Specifically, the findings highlight the promise of the reason action approach and social cognitive theory integrated with formative research in designing in-person group PA interventions for African American men living with HIV. As the population ages with increased risk of NCDs, our finding suggests that health behavior interventions need to be introduced earlier to involve younger men to accrue more extensive health benefits through increasing PA in their daily lives.
